# Active Transportation and Obesity Indicators in Adults from Latin America: ELANS Multi-Country Study

**DOI:** 10.3390/ijerph17196974

**Published:** 2020-09-24

**Authors:** Juan Guzmán Habinger, Javiera Lobos Chávez, Sandra Mahecha Matsudo, Irina Kovalskys, Georgina Gómez, Attilio Rigotti, Lilia Yadira Cortés Sanabria, Martha Cecilia Yépez García, Rossina G. Pareja, Marianella Herrera-Cuenca, Ioná Zalcman Zimberg, Viviana Guajardo, Michael Pratt, Cristian Cofre Bolados, Claudio Farías Valenzuela, Adilson Marques, Miguel Peralta, Ana Carolina B. Leme, Mauro Fisberg, André Oliveira Werneck, Danilo Rodrigues da Silva, Gerson Ferrari, on behalf of the ELANS Study Group

**Affiliations:** 1 Sports Medicine and Physical Activity Specialty, Science Faculty, Universidad Mayor, Santiago 8580745, Chile; juan.guzmanh@mayor.cl; 2 Datrics, Santiago 7500000, Chile; javiera@datrics.cl; 3 Science Faculty, Universidad Mayor, Santiago 8580745, Chile; sandra.mahecha@umayor.cl; 4 Academic Unit, MEDS Clinic, Santiago 7550000, Chile; 5 Nutrition Career, Faculty of Medical Sciences, Pontificia Universidad Católica Argentina, AAZ Buenos Aires C1107, Argentina; ikovalskys@gmail.com; 6 Department of Biochemistry, School of Medicine, Universidad de Costa Rica, San José 11501-2060, Costa Rica; georgina.gomez@ucr.ac.cr; 7 Department of Nutrition, Diabetes and Metabolism, School of Medicine, Pontificia Universidad Católica, Santiago 8330024, Chile; arigotti@med.puc.cl; 8 Department of Nutrition and Biochemistry, Pontificia Universidad Javeriana, Bogotá 110231, Colombia; ycortes@javeriana.edu.co; 9 College of Health Sciences, Universidad San Francisco de Quito, Quito 17-1200-841, Ecuador; myepez@usfq.edu.ec; 10 Nutrition Research Institute, Lima 15026, Peru; rpareja@iin.sld.pe; 11 Centro de Estudios del Desarrollo, Universidad Central de Venezuela (CENDES-UCV)/Fundación Bengoa, Caracas 1053, Venezuela; manyma@gmail.com; 12 Department of Psychobiology, Universidade Federal de São Paulo, São Paulo 04023-062, Brazil; iona.zimberg@gmail.com; 13 Nutrition, Health and Wellbeing Area, International Life Science Institute (ILSI) Argentina, Santa Fe Av. 1145, CABA C1059ABF, Argentina; viviana.guajardo@comunidad.ub.edu.ar; 14 Institute for Public Health, University of California San Diego, La Jolla, CA 92093-0021, USA; mipratt@health.ucsd.edu; 15 Laboratory of Sciences of Physical Activity, Sports and Health, Faculty of Medical Sciences, Universidad de Santiago de Chile, USACH, Santiago 7500618, Chile; cristian.cofre@usach.cl (C.C.B.); claudio.farias.v@usach.cl (C.F.V.); 16 Institute of Sports Sciences, School of Sports Sciences and Physical Activity, Universidad Santo Tomas Santiago de Chile, Santiago 8370003, Chile; 17 CIPER, Faculty of Human Motricity, Universidade de Lisboa, 1499-002 Lisbon, Portugal; amarques@fmh.ulisboa.pt (A.M.); mperalta@fmh.ulisboa.pt (M.P.); 18 ISAMB, Faculty of Medicine, Universidade de Lisboa, 1649-028 Lisbon, Portugal; 19 Department of Nutrition, School of Public Health, University of São Paulo, São Paulo 01246-904, Brazil; acarol.leme@gmail.com; 20 Department of Pediatrics, Universidade Federal de São Paulo, São Paulo 04023-061, Brazil; mauro.fisberg@gmail.com; 21 Instituto Pensi, Fundação José Luiz Egydio Setubal, Hospital Infantil Sabará, São Paulo 01227-200, Brazil; 22 Department of Nutrition, School of Public Health, Universidade de São Paulo (USP), São Paulo 01246-904, Brazil; andreowerneck@gmail.com; 23 Department of Physical Education, Federal University of Sergipe—UFS, São Cristóvão 49100-000, Brazil; danilorpsilva@gmail.com

**Keywords:** physical activity, active transportation, Latin America, obesity, body mass index, waist circumference

## Abstract

Purpose: The aim of this study was to determine the association between active transportation and obesity indicators in adults from eight Latin American countries. Methods: Data from the ELANS study, an observational multi-country study (n: 8336; 18–65 years), were used. Active transportation (walking and cycling) and leisure time physical activity was assessed using the International Physical Activity Questionnaire (long version). The obesity indicators considered were: body mass index, and waist and neck circumference. Results: In the total sample, the average time dedicated to active transportation was 24.3 min/day, with the highest amount of active transportation being Costa Rica (33.5 min/day), and the lowest being Venezuela (15.7 min/day). The countries with the highest proportion of active transportation were Ecuador (71.9%), and the lowest was Venezuela (40.5%). Results from linear regression analyses suggest that active transportation was significantly and independently associated with a lower body mass index (β: −0.033; 95% CI: −0.064; −0.002), but not with waist circumference (β: −0.037; 95% CI: −1.126; 0.390 and neck circumference (β: −0.007; 95% CI: −0.269; 0.130). Conclusions: Active transportation is significantly associated with a lower body mass index. Governments should incentivize this type of transportation as it could help to reduce the obesity pandemic in Latin America.

## 1. Introduction

Approximately 39% of the world’s adults are overweight or obese [1]. In Latin America, a prevalence of up to 60% of overweight and obesity has been reported [2], which is a considerable burden for public health. There has been a consistent association between overweight and obesity and cardiovascular diseases, diabetes, and some types of cancer [3]. Overweight and obesity and related comorbidities are a global epidemic that may be mitigated by the adoption of healthier lifestyles, including being physically active [4,5,6].

Many studies have shown that physical activity has extensive health benefits such as reducing body weight, premature mortality, and cardiovascular disease risk [5,7]. The benefits of physical activity has been extensively represented by leisure time, such as physical exercise, but evidence shows that these benefits can also be obtained through walking or cycling, as “active travel” or “active transportation” [8,9]. Active transportation has been demonstrated to be an excellent strategy for increasing physical activity level, because it is a daily behavior, an opportunity to create a healthy habit and relatively cheap alternative [10,11]. Besides, it can be integrated into everyday life particularly for those not involved or interested in engaging in leisure time physical activity [12]. Strong evidence demonstrates that physical activity related to active transportation is a crucial population-based approach, which aims to reverse the health outcomes [8,9].

Over the past two decades, the Latin American region has experienced substantial economic development due to high levels of urbanization (80%) and a unique structural, cultural and social environment, and is actually by far the most unequal region in the world [13,14]. Latin American countries are characterized by high population density patterns, the unregulated expansion of urban environments, air pollution, high crime rates, high income inequality, high levels of poverty, and population aging, which might inhibit physical activity [14,15,16]. Governments and nongovernmental organizations have been adopting several policies such as building public park facilities, cycling infrastructure and intervention strategies for improving active transportation (e.g., bike-sharing programs) to promote active transportation in Latin America [17,18,19].

Studies from Europe and the United States show that countries in which active transportation increases show decreasing obesity rates. In fact, many studies provided a contribution to the literature and filled several gaps of the association between active transportation and lower obesity indicators (body mass index) in high-income [20,21,22]. However, there is limited evidence on the association of active transportation and obesity indicators from Latin America representative samples [23]. In addition, Latin America has a high prevalence of physical inactivity and overweight/obesity [2,24]. Therefore, there is an urgent need to look for different strategies aiming to increase the active transportation levels and decrease obesity in the Latin American region. The aim of this study was to investigate the extent of the association between active transportation and obesity indicators in adults from eight Latin American countries.

## 2. Materials and Methods

### 2.1. Study Design and Sample

The Latin American Study of Nutrition and Health (Estudio Latinoamericano de Nutrición y Salud—ELANS) is a household-based cross-sectional survey that includes data collected from eight Latin American countries: Argentina, Brazil, Chile, Colombia, Costa Rica, Ecuador, Peru, and Venezuela. The study was based on a complex, multistage sample design, stratified by conglomerates, with all regions of each country represented, and random selection of main cities within each region according to the probability proportional to size method [25,26].

The sampling size required for necessary precision was calculated with a 95% confidence level and a maximum error of 3.5% and a survey design effect of 1.75. The sample was stratified by age, sex, and socioeconomic level. Socioeconomic levels were balanced based on national indexes used in each country [26]. Households within each secondary sampling unit were selected through systematic randomization. Considering quotas for age, sex and socioeconomic level, the selection of the participant belonging to the domicile was made using 50% of the sample’s next birthday, and 50% of the sample’s last birthday. Of the total sample of 9218 participants aged between 15 and 65 years, we included 8336 participants aged between 18 and 65 in this study. We excluded adolescents (15 to 17 years old) from the analyses because the ELANS study did not consider the age range of adolescents (10–19 years old) [27]. Additionally, adolescents may have restricted independent mobility that may yield different environment–physical activity associations than those observed in adults [28]. In addition, physical activity guidelines for adolescents differ from adults [29]. The rationale and design of the study is reported in more detail elsewhere [25].

All the study sites are academic-based (universities and other research institutions) and each site adhered to a common study protocol for fieldwork implementation, interviewer training, data collection and management, and the quality of control procedures. Data collection for ELANS took place from September 2014 to February 2015. The ELANS protocol was approved by the Western Institutional Review Board (#20140605) and registered at ClinicalTrials.gov (#NCT02226627). All participants of the ELANS study provided written consent.

### 2.2. Exclusion Criteria

During enrolment for the study, participants were excluded if they had a major physical/mental impairment that impacted on food intake and physical activity levels, were pregnant/lactating, and were ≤15 years old or ≥65 years old. For this study, we excluded participants <18 years of age from the total sample, and subjects without at least one anthropometric variable.

### 2.3. Active Transportation, Leisure Time and Total Physical Activity

The ELANS study employed the self-reported International Physical Activity Questionnaire-Long Form (IPAQ-LF), which was a validated measure design for Latin America [30]. IPAQ was developed to assess the physical activity levels of inhabitants from dissimilar countries [30,31]. We used a Spanish version [32] and it was adapted for all eight participating ELANS countries [14].

The ELANS study only summarized scores for active transportation and leisure time physical activity. The IPAQ-LF collected data on the reported frequency and duration (bouts at least 10 min continuously) of active transportation (walking and cycling) and leisure time physical activity (walking, moderate and vigorous physical activity) over the last seven days. Three outcomes for each domain were calculated: (1) engaging at least 10 min in active transportation and leisure time physical activity (yes, no); (2) how many days of physical activity in active transportation and leisure time physical activity; (3) how much time spent completing physical activity in active transportation and leisure time physical activity. Questions were asked separately for active transportation (walking and cycling), and leisure time physical activity (walking, moderate-intensity, and vigorous-intensity activities). Time spent in each domain was expressed as min/day.

### 2.4. Obesity Indicators

In each country, the obesity indicators (body weight, height, waist and neck circumferences) were evaluated in light clothing and without shoes using standard procedures and equipment. Data were collected by trained ELANS data collectors during a visit. Body weight (kg) and height (cm) were measured using a calibrated electronic scale Seca 213^®^ (Hamburg, Germany). The measurements were taken during the end inhalation with the participant’s head in the Frankfort plane [33]. The body mass index was calculated as the body weight divided by the square of body height (kg/m^2^). Participants were classified as underweight: <18.5; normal weight: 18.5–24.9; overweight: 25–29.9; or obese: ≥30 kg/m^2^ [34].

A nonstretchable tape with an accuracy of 0.1 cm was used to evaluate circumferences. Waist circumference was measured according to World Health Organization recommendations, i.e., with the participants standing, after regular expiration, to the nearest cm, midway between the lowest rib and the iliac crest on the horizontal plane with the subject in a standing position [34]. We used the “substantially increased risk of metabolic complications” cutoff defined by World Health Organization, >102 cm for men and >88 cm for women [34]. Neck circumference was measured in a plane as horizontal as possible, immediately below the laryngeal prominence, while standing erect with eyes facing forward [35] and was categorized as abnormal if the circumference was >39 cm for men and >35 cm for women [36]. All measurements were performed under strictly standardized conditions. Two measurements of the obesity indicators were performed, and the mean used for the analyses.

### 2.5. Sociodemographic Variables

Sociodemographics items collected by all countries included age, sex and socioeconomic level. Socioeconomic data were categorized into “low”, “medium”, and “high” based on the country specific indices [26]. Age was dichotomized into “18–34”, “35–49”, and “50–65” years. Ethnicity was classified as white, mixed (parents with dissimilar ethnicities) and other (Black, Asian, Indigenous, and Gypsy).

### 2.6. Statistical Analyses

Data were weighted considering sociodemographic variables, sex, age and socioeconomic level of the Latin American population to enable comparability for totals across countries. This was completed to have a representative sample of the adult population of each country. Descriptive statistics of mean, percentage and the associated 95% confidence interval (95% CI) were used to describe the sociodemographic obesity indicators (Table 1) and active transportation (Table 2). A Chi-square test was carried out to evaluate if there was a significant association between active transportation with the control variables mentioned before. For descriptive and categorical analysis, the self-reported time spent on active transportation was stratified into the following two categories: nonactive transportation (including those who reported nonwalking or cycling as part of their transportation) and active transportation (including those who reported ≥10 min of walking and/or cycling for transportation). A Pearson correlation was used to assess the association of active transportation with obesity indicators.

For the calculation of the CI, an asymptotic method (wald) was used based on a normal distribution. In addition, linear regression models (*β*; 95% CI) per country were used to verify the relationship of active transportation (10 min/day of active transportation as the independent variable) with body mass index, waist circumference and neck circumference (dependent variables). Our first model (model 1) was adjusted for sex, age, socioeconomic level, and ethnicity. The second model was additionally adjusted for sex, age, socioeconomic level, ethnicity, and leisure time physical activity. We also adjusted for leisure time physical activity (min/day) to examine whether the associations with time spent in active transportation were independent of time spent leisure time physical activity. All analyses were made using R-studio version 1.2.5033 [37,38]. The significance level was set at *p* < 0.05.

## 3. Results

The average response of the surveys was 99.4%, with a range between 98.5% and 99.8%, corresponding to Peru and Ecuador, respectively. The average time dedicated to active transportation was 24.3 min/day in the total sample (those who reported nonwalking or cycling as part of their transportation). Considering the full sample, the country with the highest amount of active transportation was Costa Rica (33.5 min/day), and the lowest was Venezuela (15.7 min/day). When the nonactive transportation group (those who reported nonwalking or cycling as part of their transportation) was excluded, the average was 41.6 min/day. Costa Rica still had the highest amount of active transportation (50.3 min/day), while Venezuela had the shortest time (35.6 min/day). The average time spent on leisure time physical activity was 16.6 min/day (95% CI: 15.3; 17.9) for the group that did not engage in active transportation. Meanwhile, for the group that does at least 10 min of active transportation, it was 35.2 min/day (95% CI: 33.3; 37.1) (*p* < 0.001).

Table 1 shows a descriptive analysis of the sample, dividing the sample into those who did not complete active transportation from those who did. There was a significant association between country and active transportation (*p* < 0.001). The country with the highest proportion of active transportation was Ecuador (71.9%), and the lowest was Venezuela (40.6%). There were no statistically significant differences in the proportion of active transportation between men and women, age groups, or socioeconomic level. On the other hand, the white ethnic group had a lower proportion of active transportation than the other groups (*p* < 0.001). When comparing the proportion of active transportation by body mass index, it was observed that the prevalence of active transportation was lower among overweight and obese participants than those with normal body mass index (56.1% and 51.4% vs. 57.3%) (*p* < 0.001). The same difference is observed for waist and neck circumference, where a lower proportion of active transportation was observed among those with increased indicators compared to those with a normal waist and neck circumference (56.5% vs. 52.6% and 56.3% vs. 53.0%, respectively) (*p* < 0.001).

The correlation between the active transportation and obesity indicator (body mass index, waist and neck circumference) by country is shown in Table 2. Body mass index and waist circumference were weakly and negatively associated with all countries (except Ecuador); however, the highest correlation value was Argentina and Venezuela. Furthermore, there was a weak and negative association between active transportation and neck circumference in the full sample—Peru and Venezuela (*p* < 0.05). The highest correlation value was Venezuela. Active transportation was not associated with neck circumference in Argentina, Brazil, Chile, Colombia, Costa Rica, and Ecuador.

We also conducted two different specifications for every regression model to investigate the association between active transportation and obesity indicators as continuous variables (Table 3). The first model, adjusted for sex, age, socioeconomic level, and ethnicity, showed that active transportation was significantly associated with a lower body mass index and waist circumference, but not with neck circumference. In the second model, including leisure time physical activity, active transportation was significantly associated only with a lower body mass index. An increase of 10 min regarding active transportation per day resulted in a −0.033 Kg/m^2^ change in the body mass index. No statistical association was found between active transportation with waist and neck circumference.

## 4. Discussion

This is the first multi-country study with a representative sample of Latin Americans to determine the extent of association between active transportation and obesity indicators in adults. In general, we demonstrated that a greater amount of time on active transportation was associated with a lower body mass index. This association was independent of age, sex, socioeconomic level, ethnicity and leisure time physical activity. These are novel results that reflect what has been reported in some countries and multinational studies [21,39].

Our findings are compatible with other studies that reported a lower body mass index associated with active transportation [40,41]. Flint et al. [42] studied the association of active commuting and body mass index in a cross-sectional study of more than 15,000 people in the United Kingdom. Active commuting was associated with a lower body mass index—0.97 Kg/m^2^ and 0.87 Kg/m^2^ in men and women, respectively. Later, the same author [21] showed similar results in a cross-sectional study of more than 264,000 middle-aged adults. They found a lower body mass index—1.0 Kg/m^2^ and 0.67 Kg/m^2^ in men and women, respectively. Laverty et al. [39] carried out a multinational study of six middle-income countries, with more than 40,000 participants. They found that ≥150 min/week of active transportation was associated with a lower body mass index of 0.54 Kg/m^2^. Wojan et al. [43] searched for this association in the United States in a study of 12,405 adults. They found that active commuting produced an average treatment effect of −1.83 Kg/m^2^.

Different from previous studies [39,41], we did not find an association between active transportation and waist circumference. This was observed when the analysis was adjusted for leisure time physical activity (model 2), indicating a potential co-existence of physical activity in these different contexts and reinforcing a stronger association of leisure time physical activity with waist circumference compared to transport physical activity. Concerning neck circumference, although studies have found associations with moderate to vigorous physical activity [44], their relationship with active transportation in adults has not been describe before. Here, we observed that active transportation was associated with a lower neck circumference in Costa Rica and Peru only, which can be related to the higher prevalence of active transportation in these countries. However, further studies are needed in order to explore specific amounts and intensities of transport physical activity associated with obesity indicators.

We found that the mean time spent in active transportation was 24.3 min/day. Latin America has low levels of active transportation compared to European countries, such as Germany or Sweden [45,46,47]. Only about one-fifth of the time for transportation is spent in active transportation in Latin America [13]. This is especially important considering that 27% to 47% of people in Latin America do not achieve the physical activity recommendations for health [47]. In a previous study, we showed that the prevalence of physical inactivity (<150 min/week) was 40.6% (95% CI: 38.8, 42.5); ranging from 26.9% (Chile) to 47% (Costa Rica and Venezuela). Physical inactivity was greater in Argentina (43.7%; 95% CI: 38.1, 49.5), Brazil (43.5%; 95% CI: 39.4, 47.7), Costa Rica (48.0%; 95% CI: 42.1, 53.9) and Venezuela (47.1%; 95% CI: 42.1, 52.3) [47]. In this context, promoting public policies aimed to encourage this type of transport, as it can help more people achieve the physical activity recommendation for health [43]. This could help to significantly decrease the disease burden associated with physical inactivity [5,48]. Other countries have estimated that moderate increases in active transportation lead to savings in national public health services. For example, in the United Kingdom, it was found that an increase of 1 km in walking and 3 km in cycling per day would save £17 billion for the national health system in 20 years [49].

Moreover, those who change their mode of commuting gain weight if they shift from cycling or walking to car travel, but lose weight when shifting from cars to active commuting [50]. Martin et al. [40] showed that changing from private transport to active commuting decreased body mass index by 0.32 Kg/m^2^. Conversely, shifting from active commuting to private transport increased body mass index by 0.34 Kg/m^2^. As established before, urban factors impact on the choice of transport modality [13,23]. One of the strategies to increase active transportation in the population is to invest in creating neighborhoods and cities that favor this type of transport [18,23]. This could be a key to reducing the obesity pandemic that exists in Latin America and the world. Several countries in this region have invested in infrastructure to encourage the use of active transportation—as is the case of some cities, such as Bogotá in Colombia, which invested in creating exclusive bike paths [18]. Our results are consistent with the findings reported in high-income countries. Therefore, they are generalizable to some extent for other middle-income countries.

The strengths of this study include the large sample from eight Latin American countries and standardized objective anthropometric indicators (body mass index, waist circumference and neck circumference). Given that a representative sample of these countries was studied, these results can be extrapolated to the rest of the adult population of these eight Latin American countries. Waist circumference is a well-established indicator of central obesity, a key factor in cardiovascular disease risk and metabolic syndrome [51]. While neck circumference is an indicator, which is still under study [36,52,53], its relationship with active transportation had not been previously reported in adults.

Within the limitations, we found that part of the sample (418 participants, 5.01%) was not classified by ethnicity. When using a subjective active transport measurement (IPAQ), there may be reporting bias [54]. From the four domains of physical activity, only the active transportation and leisure time physical activity sections were included. This decision was made for two reasons: (i) due to the greater relevance of these domains for targeted physical activity programming and policy efforts [30] and (ii) the weak validity of occupational and home-based physical activity questions [55]. The cross-sectional design of this study limits generalizability and limits conclusions regarding of causality. There is a possibility of reverse causality and residual confusion; it could be that obese people less actively travel.

## 5. Conclusions

This study supports that active transportation is significantly associated with a lower body mass index. Incentive active transportation could help to reduce the obesity pandemic and help more people to meet physical activity recommendations in Latin America. These findings are consistent with existing evidence from other regions of the world. For these reasons, the generation of public policies aiming to promote active transportation in Latin America should be prioritized.

## Figures and Tables

**Table 1:** Sociodemographic and obesity indicators of the population according to active transportation categories.

Variable and Category	Nonactive Transportation (%) (<10 min of Walking and/or Cycling)	Active Transportation (%) (≥10 min of Walking and/or Cycling)
*n* (%)	3733 (44.8)	4603 (55.2)
Country		
Argentina	565 (48.8) ***	592 (51.2) ***
Brazil	889 (48.6) ***	941 (51.4) ***
Chile	353 (44.3) ***	443 (55.7) ***
Colombia	482 (42.7) ***	646 (57.3) ***
Costa Rica	255 (35.7) ***	460 (64.3) ***
Ecuador	195 (28.1) ***	500 (71.9) ***
Peru	390 (39.0) ***	609 (61.0) ***
Venezuela	604 (59.5) ***	412 (40.5) ***
Sex		
Men	1744 (44.5)	2176 (55.5)
Women	1989 (45.0)	2427 (55.0)
Age (years)		
18–34	1733 (44.0)	2203 (56.0)
35–49	1179 (45.9)	1388 (54.1)
50–65	821 (44.8)	1012 (55.2)
Socioeconomic level		
Low	1953 (45.1)	2375 (54.9)
Medium	1428 (44.5)	1782 (55.5)
High	352 (44.1)	446 (55.9)
Ethnicity		
White	1442 (49.5) ***	1470 (50.5) ***
Mixed	1764 (42.1) ***	2429 (57.9) ***
Other	341 (41.9) ***	472 (58.1) ***
Body mass index (Kg/m^2^)		
<18.5	99 (45.8) ***	117 (54.2) ***
18.5–24.9	1249 (42.7) ***	1679 (57.3) ***
25–29.9	1305 (43.9) ***	1665 (56.1) ***
≥30	1079 (48.6) ***	1143 (51.4) ***
Waist circumference (cm)		
≤102 (M) or 88 (W)	2408 (43.5) ***	3132 (56.5) ***
>102 (M) or 88 (W)	1324 (47.4) ***	1472 (52.6) ***
Neck circumference (cm)		
≤39 (M) or 35 (W)	2430 (43.7) **	3132 (56.3) **
>39 (M) or 35 (W)	1303 (47.0) **	1471 (53.0) **

Figures as percentages if not stated otherwise. Chi-square tests were used for distributions. ** *p* ≤ 0.01; *** *p* ≤ 0.001. M: men; W: women.

**Table 2:** Correlation between active transportation and obesity indicators per country.

Country	Body Mass Index (Kg/m^2^)		Waist Circumference (cm)		Neck Circumference (cm)	
	r	*p*-Value	r	*p*-Value	r	*p*-Value
Full sample	−0.196	<0.001	−0.199	<0.001	−0.121	0.048
Argentina	−0.235	<0.001	−0.194	<0.001	−0.104	0.901
Brazil	−0.155	0.015	−0.182	<0.001	0.113	0.555
Chile	−0.184	0.015	−0.200	0.004	0.128	0.413
Colombia	−0.227	<0.001	−0.195	<0.001	−0.129	0.328
Costa Rica	−0.221	<0.001	−0.251	<0.001	−0.159	0.099
Ecuador	−0.138	0.302	−0.158	0.118	−0.114	0.702
Peru	−0.217	<0.001	−0.202	<0.001	−0.168	0.027
Venezuela	−0.231	<0.001	−0.244	<0.001	−0.184	0.006

**Table 3:** Linear regression models for association of active transportation with obesity indicators.

	Model 1						Model 2					
Country	Body Mass Index (Kg/m^2^)		Waist Circumference (cm)		Neck Circumference (cm)		Body Mass Index (Kg/m^2^)		Waist Circumference (cm)		Neck Circumference (cm)	
	β (95% CI)	*p*-Value	β (95% CI)	*p*-Value	β (95% CI)	*p*-Value	β (95% CI)	*p*-Value	β (95% CI)	*p*-Value	β (95% CI)	*p*-Value
Full sample	−0.044 (−0.074; −0.014)	0.004	−0.077 (−0.150; −0.003)	0.041	−0.013 (−0.032; 0.007)	0.206	−0.033 (−0.064; −0.002)	0.036	−0.037 (−1.126; 0.390)	0.341	−0.007 (−0.269; 0.130)	0.496
Argentina	−0.074 (−0.148; 0.006)	0.059	−0.036 (−0.232; 0.160)	0.717	0.054 (0.006; 0.098)	0.023	−0.046 (−1.254; 0.333)	0.255	−0.101 (−1.538; 2.518)	0.468	0.060 (−0.123; 1.082)	0.064
Brazil	−0.030 (−0.101; 0.041)	0.407	−0.059 (−0.236; 0.119)	0.515	0.001 (−0.050; 0.053)	0.969	−0.038 (−1.116; 0.357)	0.313	−0.034 (−2.172; 1.496)	0.718	−0.006 (−0.600; 0.474)	0.818
Chile	−0.012 (−0.111; 0.088)	0.821	−0.058 (−0.306; 0.190)	0.646	0.009 (−0.049; 0.068)	0.752	−0.006 (−1.085; 0.964)	0.908	−0.058 (−3.151; 1.986)	0.656	0.014 (−0.469; 0.740)	0.661
Colombia	−0.071 (−0.137; −0.003)	0.038	−0.162 (−0.323; 0.007)	0.053	−0.025 (−0.065; 0.016)	0.230	−0.06 (−1.302; 0.096)	0.091	−0.138 (−3.093; 0.327)	0.113	−0.012 (−0.536; 0.304)	0.588
Costa Rica	−0.081 (−0.172; 0.012)	0.083	−0.129 (−0.352; 0.099)	0.262	−0.056 (−0.106; −0.007)	0.027	−0.072 (−1.658; 0.216)	0.131	−0.097 (−3.271; 1.326)	0.407	−0.054 (−1.043; −0.032)	0.037
Ecuador	0.043 (−0.054; 0.139)	0.381	−0.018 (−0.229; 0.192)	0.868	−0.025 (−0.085; 0.034)	0.407	0.055 (−0.450; 1.553)	0.006	0.021 (−1.961; 2.380)	0.850	−0.020 (−0.82; 0.415)	0.520
Peru	−0.025 (−0.111; 0.061)	0.563	−0.088 (−0.292; 0.117)	0.403	−0.070 (−0.123; −0.017)	0.009	−0.017 (−1.066; 0.718)	0.702	−0.051 (−2.636; 1.612)	0.636	−0.067 (−1.221; −0.128)	0.016
Venezuela	−0.043 (−0.161; 0.068)	0.463	−0.141 (−0.430; 0.113)	0.308	−0.058 (−0.134; 0.010)	0.114	−0.029 (−1.445; 0.859)	0.618	−0.101 (−3.737; 1.720)	0.468	−0.049 (−1.215; 0.240)	0.189

Model 1: adjusted for sex, age, socioeconomic level, and ethnicity. Model 2: Model 1 + leisure time physical activity.
